# Homeotic Gene *teashirt* (*tsh*) Has a Neuroprotective Function in Amyloid-Beta 42 Mediated Neurodegeneration

**DOI:** 10.1371/journal.pone.0080829

**Published:** 2013-11-25

**Authors:** Michael T. Moran, Meghana Tare, Madhuri Kango-Singh, Amit Singh

**Affiliations:** 1 Department of Biology, University of Dayton, Dayton, Ohio, United States of America; 2 Premedical Program, University of Dayton, Dayton, Ohio, United States of America; 3 Center for Tissue Regeneration and Engineering at Dayton (TREND), University of Dayton, Dayton, Ohio, United States of America; Roswell Park Cancer Institute, United States of America

## Abstract

**Background:**

Alzheimer's disease (AD) is a debilitating age related progressive neurodegenerative disorder characterized by the loss of cognition, and eventual death of the affected individual. One of the major causes of AD is the accumulation of Amyloid-beta 42 (Aβ42) polypeptides formed by the improper cleavage of amyloid precursor protein (APP) in the brain. These plaques disrupt normal cellular processes through oxidative stress and aberrant signaling resulting in the loss of synaptic activity and death of the neurons. However, the detailed genetic mechanism(s) responsible for this neurodegeneration still remain elusive.

**Methodology/ Principle Findings:**

We have generated a transgenic *Drosophila* eye model where high levels of human Aβ42 is misexpressed in the differentiating photoreceptor neurons of the developing eye, which phenocopy Alzheimer's like neuropathology in the neural retina. We have utilized this model for a gain of function screen using members of various signaling pathways involved in the development of the fly eye to identify downstream targets or modifiers of Aβ42 mediated neurodegeneration. We have identified the homeotic gene *teashirt* (*tsh*) as a suppressor of the Aβ42 mediated neurodegenerative phenotype. Targeted misexpression of *tsh* with Aβ42 in the differentiating retina can significantly rescue neurodegeneration by blocking cell death. We found that Tsh protein is absent/ downregulated in the neural retina at this stage. The structure function analysis revealed that the PLDLS domain of Tsh acts as an inhibitor of the neuroprotective function of *tsh* in the *Drosophila* eye model. Lastly, we found that the *tsh* paralog, *tiptop* (*tio*) can also rescue Aβ42 mediated neurodegeneration.

**Conclusions/Significance:**

We have identified *tsh* and *tio* as new genetic modifiers of Aβ42 mediated neurodegeneration. Our studies demonstrate a novel neuroprotective function of *tsh* and its paralog *tio* in Aβ42 mediated neurodegeneration. The neuroprotective function of *tsh* is independent of its role in retinal determination.

## Introduction

Alzheimer's disease (AD; OMIM: 104300), first described more than 100 years ago, is an age related, progressive neurodegenerative disorder characterized by the loss of neurons in the hippocampus and cortex. It results in the loss of cognition and memory and eventually leads to the death of the affected individual [1-3]. AD can be hereditary or acquired. Familial forms of AD have been associated with mutations in the amyloid precursor protein (APP). Among the multiple causes for AD that have been identified, the accumulation of amyloid plaques around neurons in the brain and the generation of neurofibrillary tangles (NFTs) due to hyperphosphorylation of microtubule binding protein Tau [4,5], are the two major causes for manifestation of AD. The amyloid plaques are formed by improper cleavage of the trans-membrane protein APP. APP is proteolytically processed in the extracellular and intracellular domains by β and then γ-secretase enzymes [2-6], which leads to the generation of a cytoplasmic fragment that has been implicated in intracellular and nuclear signaling. Normally, APP cleavage results in a forty amino acid long polypeptide (Aβ40), however, improper cleavage of APP results in a forty two amino acid long polypeptide, and hence called as amyloid-beta 42 (Aβ42). These extra two amino acids cause the molecule to become hydrophobic, resulting in the formation of amyloid plaques [2-5]. The "amyloid hypothesis" suggests that these Aβ42 plaques are toxic in nature and trigger aberrant signaling and disruption of normal cellular processes inside the neuronal cell which lead to loss of synaptic function and death of the neuron [2-4,6]. Thus, the accumulation of plaques is responsible for the gradual decline of mental cognition, awareness, and eventual death, of patients who suffer from AD. Therefore, it is important to understand how Aβ42 plaques trigger neurotoxicity and cell death in AD. The genetic mechanism behind the onset of this disease has not been fully understood. Several animal models, like the mouse, fly [7-10] etc., have been developed to understand the genetic underpinnings of this disease as the genetic machinery is conserved from insects to humans. 

*Drosophila melanogaster*, fruit fly, because of its shorter life cycle, highly conserved signaling pathways, and less functional redundancy has proved to be an important complementary animal model for human diseases. The information generated in fly model system can be extrapolated and tested in mammalian model systems [7,11]. The *Drosophila* eye has been extensively used to model human neurodegenerative disorders [6,10,12-16] as important signaling pathways required for the proper development and differentiation of the fly visual system are highly conserved with that of vertebrates [8]. *Drosophila* , a holometabolous insect, has a blue print for its adult organs housed inside the larvae referred to as the imaginal discs. The larval eye-antennal imaginal disc is a complex disc, which gives rise to the adult compound eye, antenna and head upon differentiation [17-20]. The retinal precursor cells in the eye imaginal disc undergo differentiation to form the photoreceptor neurons in the adult eye [21-23]. Retinal differentiation begins as a synchronous wave from the posterior margin of the eye imaginal disc and proceeds towards the anterior margin of the eye and is referred to as the Morphogenetic Furrow (MF) [21]. The MF leaves behind the differentiated photoreceptor neurons. Eight photoreceptor neurons and several support cells form a unit eye called as the ommatidium. The compound eye of the adult fly is comprised of about 800 ommatidia. In the pupal retina, the excessive cells other than the differentiated cells are eliminated by programmed cell death (PCD) [24]. There is no PCD during earlier stages of larval eye development. However, abnormal extracellular signaling due to inappropriate levels of morphogens may trigger cell death in the developing larval eye imaginal disc [25].

We have utilized the *Drosophila* eye to model AD [26]. Using a targeted misexpression approach [27], we misexpressed higher levels of human Aβ42 gene in the differentiating photoreceptors of the developing eye using a Glass Multiple Repeat (GMR) Gal4 driver [28]. Misexpression of human Aβ42 in the differentiating photoreceptor neurons of the fly retina exhibits the progressive neurodegenerative phenotypes which mimic the neuropathology of AD patients [26]. In order to discern the genetic basis of Aβ42 mediated neurodegeneration, we have employed our transgenic fly model to analyze the toxic effect of Aβ42 accumulation on signaling pathways through a forward genetic screen. These pathways include the core retinal determination (RD) pathway, which is made up of a cascade of genes *viz*., PAX-6 homolog *eyeless* ** *(* *ey* *)*, *eyes* ** *absent* (*eya*)*,* ** *sine* ** *oculis* (*so*) and *dachshund* ** *(* *dac* *)*, which are responsible for the initiation and differentiation of eye development [18-20,29,30]. We also tested negative regulators of eye development like *homothorax* (*hth*) [31,32]. *hth* is known to suppress retinal differentiation in the eye and promote the head specific fate [33]. Another negative regulator of eye development is Wingless (Wg), a member of the highly conserved Wnt/ Wingless signaling pathway, which is responsible for regulating early growth, dorso-ventral (DV) lineage and, during the latter part of development, restricting the eye fate on outer dorso-ventral margins of the developing eye [34,35]. Wnt signaling is antagonized by another highly conserved TGF beta (TGFβ) signaling pathway, referred to as Decapentaplegic (Dpp) signaling in *Drosophila* [36-38]. Dpp signaling collaborates with Hedgehog (Hh) signaling to promote retinal differentiation in the developing eye as well as antagonize Wg signaling [39]. Another signaling pathway tested includes Notch (N) signaling pathway. N is a transmembrane receptor activated by "DSL" class ligands which is involved in: cell proliferation and differentiation during eye development, setting up the dorsal-ventral compartment boundary, planar polarity and spacing of the ommatidial clusters, and in cell fate specification [40]. In the developing eye imaginal disc, N activity is highest at the equator, the boundary between the dorsal and ventral halves of the eye. N pathway also employs the same secretases which are involved in the processing of APP for the cleavage of the protein [41]. We tested components of all these pathways in our forward genetic screen based on a premise similar to our earlier reported gain-of-function screen in the developing *Drosophila* eye [42].

We have identified *teashirt* (*tsh*) as a modifier of Aβ42 mediated neurodegeneration. A homeotic gene, *tsh* encodes a zinc finger transcription factor. The full length Tsh protein is 954 amino acids long and has three DNA binding zinc finger domains and a N-terminal PLDLS domain that is required for interaction with CtBP protein [31,43-47]. In an enhancer trap screen for genes involved in embryonic development, *tsh* was first identified and assigned a role in specifying trunk identity during embryogenesis through its interactions with the Hox protein network [45,47]. However, *tsh* is also known to play additional roles in other tissues. The role of *tsh* in eye development was first reported in an enhancer trap screen for genes exhibiting domain specific expression of the mini-white reporter gene during eye development [31]. In the developing *Drosophila* eye imaginal disc, *tsh* exhibits domain specific function by promoting growth in the dorsal eye, whereas it suppresses eye fate in the ventral by ectopically activating Wg signaling [19,46,48]. *tsh* has also been shown to induce ectopic eyes [31], while its zinc fingers and PLDLS binding motif have been shown to be involved in its ectopic eye formation function [49]. The paralog of *tsh*, *tiptop* (*tio*) [50], encodes a 1025 amino acids long C_2_H_2_ zinc finger protein of the Teashirt family [47]. It has been shown that *tio* and *tsh* have similar expression patterns in the eye [44,47,49]. It has also been suggested that *tio* exhibits functional redundancy with *tsh* through a mutual repression mechanism [43,44]. 

Here we report a novel neuroprotective function of *tsh* in Aβ42 mediated neurodegeneration. We demonstrate that upregulation of full length Tsh expression can significantly rescue the Aβ42 mediated neurodegenerative phenotype by blocking cell death of neurons. The structure function analysis determined that the PLDLS domain of Tsh acts as a suppressor of its neuroprotective function in Aβ42 mediated neurodegeneration. We have identified *tio*, a paralog of *tsh* [50], which can also rescue Aβ42 mediated neurodegeneration. Furthermore, the structure function analysis determined that the Zn4 and PLDLS domains of *tio* act as suppressors of the neuroprotective function of *tio* in Aβ42 induced neurodegeneration. 

## Materials and Methods

### Fly Stocks

All fly stocks used in this study are listed and described in Flybase (http:// flybase.bio.indiana.edu). The fly stocks used in this study were UAS*-ey* [29], UAS*-eya* [30], UAS*-so*, UAS-*eya* *;* ** *so* [51], UAS*-dac* [52], UAS*-wg* [53], UAS*-sgg* [54], UAS-N [55], UAS*-dpp* [56], UAS*-omb* [57], UAS-*ptc* [58]. The fly stocks used for *homothorax* are UAS-EN-HTH^1-430^ or UAS-EN-Hth^ENR^, a dominant negative allele of *hth*, generated by fusing the *Drosophila* Engrailed repression domain [59] to a truncated form of Hth (amino acids1-430)[60], and a UAS transgene harboring the full length *hth*, (*h* *t* *h*-FL) [61,62]. Other stock used were GMR-Gal4, UAS-Aβ42 [26] and a *tsh* ** *lacZ* reporter transgene, *tshA8* [63]. Various *tsh* constructs used in this study were UAS*-tsh* (full length) [64], UAS-*tsh∆Zn1*, UAS-*tsh∆Zn2*, UAS-*tsh∆Zn3*, UAS*-tsh/tioZn4*, UAS*-tsh∆PLDLS*. The *tsh∆Zn1* lacks amino acid residue 356-378, UAS-*tsh∆Zn2* lacks amino acid residue 478-490, UAS-*tsh∆Zn3* lacks amino acid residue 535-557. The UAS*-tsh∆PLDLS* lacks amino acid residue 188-192 in the N-terminal region, where the CtBP binding site is deleted [49]. The various *tio* constructs used in this study are UAS*-tio* [47] UAS*-tio∆Zn1*, UAS*-tio∆Zn2*, UAS*-tio∆Zn3*, UAS-*tio∆Zn4*, UAS*-tio∆PLDLS* [49]. The various truncated constructs of *tio* lacked amino-acid residues *tio∆Zn1* (319-341a.a.), UAS-*tio∆Zn2* (428-450 a.a.), UAS-*tio∆Zn3* (501-523 a.a.), and UAS-*tio∆Zn4* (928-949 a.a.). The UAS*-tio∆PLDLS* lacks 187-191 amino acid residues in N-terminal region, where the CtBP binding site is deleted [49].

We have employed a Gal4/UAS system for targeted misexpression studies [27]. All Gal4/UAS crosses were maintained at 18°C, 25°C and 29°C, unless specified, to sample different induction levels. The adult flies were maintained at 25°C, while the cultures after egg laying (progeny) were transferred to 29°C for further growth. The misexpression of Aβ42 in the differentiating retina (GMRGal4>UAS-Aβ42) exhibits a stronger neurodegenerative phenotype at 29°C with no penetrance [26]. All the targeted misexpression experiments were conducted using the Glass Multiple Repeat driver line (GMR-Gal4) which directs expression of transgenes in the differentiating retinal precursor cells of the developing eye imaginal disc and pupal retina [28].

### Immunohistochemistry

 Eye-antennal imaginal discs were dissected from wandering third-instar larvae in 1X PBS and stained following the standard protocol [46]. Eye-imaginal discs were fixed in 4% paraformaldehyde and stained with a combination of antibodies using the standard protocol. Primary antibodies used were rabbit anti-Tsh (1:150, a gift from Stephen Cohen), rat anti-Elav (1:50; Developmental Studies Hybridoma Bank, DSHB), mouse anti-Dlg (1:100; DSHB), mouse anti 22C10 (1:100; DSHB), mouse anti-Chaoptin (MAb24B10) (1:100; DSHB) [65], mouse anti-β-galactosidase (1:100; DSHB). Secondary antibodies (Jackson Laboratories) used consisted of donkey anti-rabbit IgG conjugated with FITC (1:200), donkey anti-mouse IgG conjugated with Cy3 (1:250), and goat anti-rat IgG conjugated with Cy5 (1:250). The tissues were mounted in vectashield (Vector labs) and all immunofluorescence images were captured using the Olympus Fluoview 1000 Laser Scanning Confocal Microscope. The final images and figures were prepared using Adobe Photoshop CS4 software. 

### Detection of Cell Death

Cell death was detected using TUNEL assays [26,66-68]. TUNEL assays were used to identify the cells undergoing cell death where the cleavage of double and single stranded DNA is labeled by a Fluorescein. The fluorescently labeled nucleotides are added to 3' OH ends in a template-independent manner by Terminal Deoxynucleotidyl Transferase (TdT). The fluorescent label tagged fragmented DNA within a dying cell can be detected by fluorescence or confocal microscopy. Eye-antennal discs after secondary antibody staining [69] were blocked in 10% normal donkey serum in phosphate buffered saline with 0.2% Triton X-100 (PBT) and labeled for TUNEL assays using a cell death detection kit from Roche Diagnostics. 

The TUNEL positive cells were counted from five sets of imaginal discs and were used for statistical analysis using Microsoft Excel 2010. The P-values were calculated using one-tailed *t*-test and the error bars represent Standard Deviation from Mean [26].

### Scanning Electron Microscopy

 All the flies were prepared through a series of increasing acetone concentration treatments following the standard protocol [70]. Each sample was then treated in 1:1 acteone/HMDS (Hexa Methyl Di Silazane, Electron Microscopy Sciences) solution for 24 hours. This was followed by treatment with 100% HMDS and allowed to air dry in the hood. Dehydrated flies were mounted on Electron microscopy stubs and coated with gold using a Denton vacuum sputter coater. Images were captured using a Hitachi S-4800 High Resolution Scanning Electron Microscope (HRSEM). The final images and figures were prepared using Adobe Photoshop CS4 software.

### Adult Eye Imaging

 Adult flies were prepared for imaging by freezing at -20ºC for approximately 2 hours followed by mounting the fly on a dissection needle. The needle with fly was suspended horizontally over a glass slide using molding putty. Images were captured on a MrC5 color camera mounted on an Axioimager.Z1 Zeiss Apotome using Z-sectioning approach. Final images were generated by compiling the individual stacks from the Z-sectioning approach using the extended depth of focus function of Axiovision software version 4.6.3. 

## Results

### Identification of genetic modifiers of Aβ42 mediated neurodegeneration

**Figure 1:**
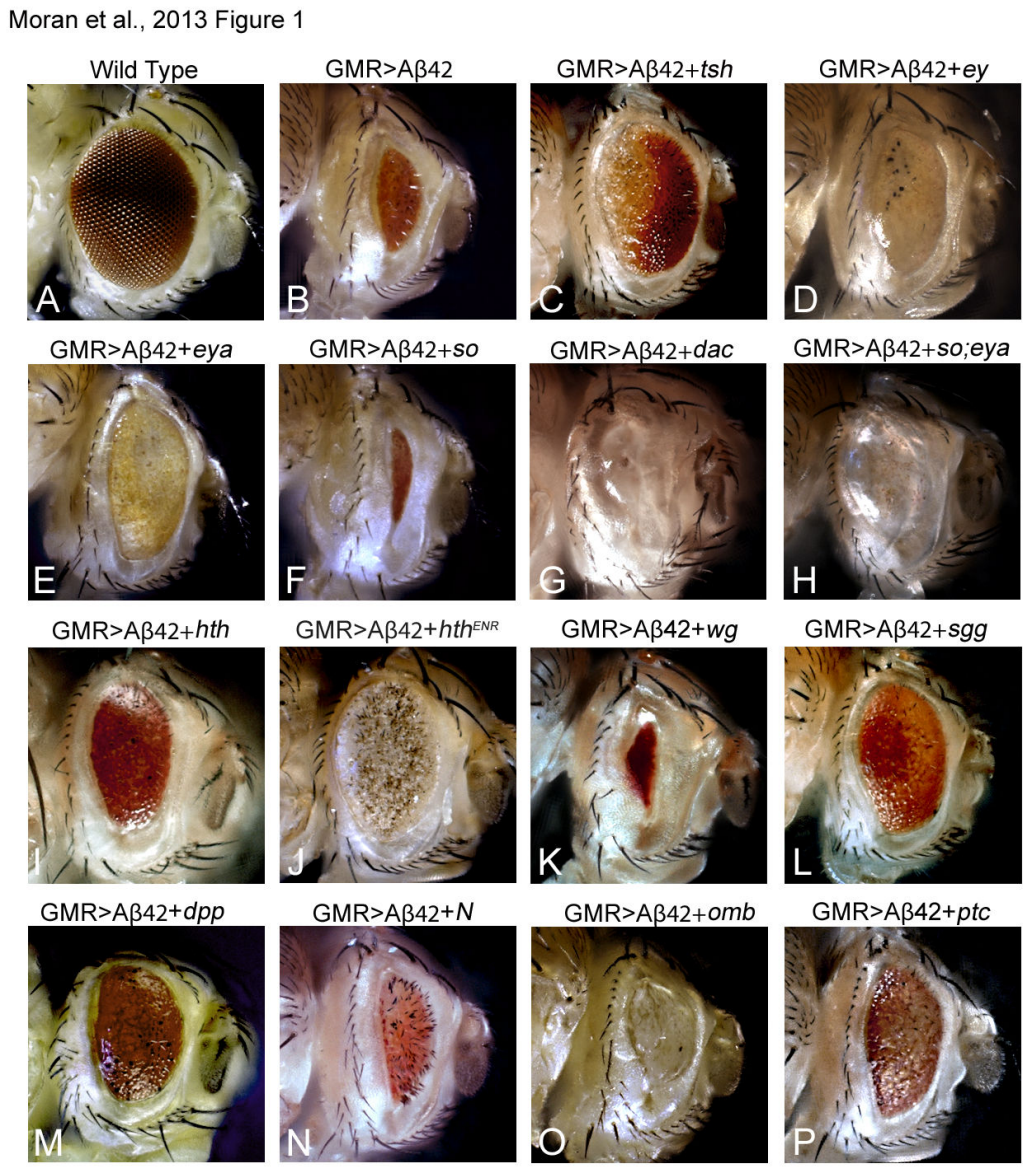
Genetic modifiers of amyloid-beta 42 (Aβ42) mediated neurodegeneration in the *Drosophila* eye. In a gain-of-function forward genetic screen, the candidate genes of interest were misexpressed along with Aβ42 in the differentiating neurons of the developing eye. The effect of upregulation of the gene of interest on the Aβ42 mediated neurodegenerative phenotype is assayed in the adult eye. In comparison to the (a) wild-type compound eye, (B) misexpression of Aβ42 (GMR>Aβ42) results in strong neurodegeneration in the adult eye, as evident from the highly reduced size, glazed appearance and fusion of ommatidia. However, targeted misexpression of (C) *teashirt* (tsh) with Aβ42 (GMR>Aβ42+*t* *s* *h*) results in a significant rescue of the neurodegenerative phenotype. (D-J) Targeted misexpression of Aβ42 along with the retinal determination genes (D) eyeless (ey), (GMR>Aβ42+*e* *y*), (E) eyes absent (eya), (GMR>Aβ42+*e* *y* *a*), (F) sine *oculis* (so), (GMR>Aβ42+*s* *o*), (G) *dacshund* (dac), (GMR>Aβ42+*d* *a* *c*), and (H) *eya* and so (GMR>Aβ42+*e* *y* *a*+*s* *o*) did not show any significant rescue of the Aβ42 (GMR>Aβ42) mediated neurodegenerative phenotype. Even though (D) GMR>Aβ42+*e* *y* and (E) GMR>Aβ42+*e* *y* *a* cause a subtle increase in the eye field but the neurodegenerative phenotype is not rescued. Furthermore, black necrotic spots are also seen suggesting that members of the core retinal determination genes machinery cannot rescue Aβ42 (GMR> Aβ42) mediated neurodegeneration. Targeted misexpression of negative regulator of eye development (D) *homothorax* (hth), (GMR>Aβ42+*h* *t* *h*), (E) dominant negative *hth* (hth^ENR^), (GMR>Aβ42+*h* *t* *h* ^*ENR*^) also did not affect Aβ42 mediated neurodegeneration. Other signaling pathways tested in the gain-of-function screen were (K, L) Wingless (Wg), (M) Decapentaplegic (Dpp), (N) Notch (N) and (P) Hedgehog (Hh). (K) Upregulation of Wg (GMR>Aβ42+*w* *g*) enhances whereas (L) downregulating Wg signaling by using an antagonist of Wg signaling shaggy (GMR>Aβ42+*s* *g* *g*) can significantly rescue Aβ42 mediated neurodegeneration. (M) Activation of Dpp signaling (GMR>Aβ42+*d* *p* *p*) can significantly rescue Aβ42 mediated neurodegeneration. However upregulation of (N) *N* (GMR>Aβ42+*N*), (O) optomotor blind (omb), (GMR>Aβ42+*o* *m* *b*) and (P) patched (GMR>Aβ42+*p* *t* *c*) did not rescue the neurodegenerative phenotype. The magnification of all brightfield images of the adult is 10X.

We have generated a transgenic fly model where GMR enhancer drives the expression of the human Aβ42 gene (GMR>Aβ42) in differentiating photoreceptor neurons of the developing retina [26,28]. Accumulation of Aβ42 triggers aberrant signaling mechanism(s) and impairs the basic cellular processes leading to the death of neurons in the developing neural retina of pupa and the adult eye ( Figure 1, Table 1) [26]. However, the genetic basis of Aβ42 mediated neurodegeneration has not been fully understood. We performed a forward gain-of-function genetic modifier screen using a candidate gene approach [42] to identify the downstream targets or genetic modifiers of Aβ42 mediated neuropathy in the developing *Drosophila* eye. We looked for modifiers of the GMR>Aβ42 phenotype when we individually misexpress the member genes of various highly conserved signaling pathways (Figure 1, Table 1). The premise of the screen was based on the observation that the neurodegenerative phenotypes of the GMR>Aβ42 exhibits no penetrance. Therefore, any deviation in this phenotype can be attributed to misexpression of the gene(s) of interest. 

**Table 1:** Summary of the effect of targeted misexpression of genetic modifiers on the Aβ42 mediated neurodegenerative phenotype.

No.	Genotype	Phenotype
1.	Wild-Type	+ + + + +
2.	GMR>Aβ42	- - -
3.	GMR>Aβ42+*tsh*	+ + +
4.	GMR>Aβ42+*ey*	- -
5.	GMR>Aβ42+*eya*	-
6.	GMR>Aβ42+*so*	- - - -
7.	GMR>Aβ42+*so;eya*	- -
8.	GMR>Aβ42+*dac*	- - - - -
9.	GMR>Aβ42+*hth*	-
10.	GMR>Aβ42+*hth* ^*ENR*^	+
11.	GMR>Aβ42+*wg*	- - - -
12.	GMR>Aβ42+*sgg*	+ +
13.	GMR>Aβ42+*N*	- -
14.	GMR>Aβ42+*dpp*	+ +
15.	GMR>Aβ42+*omb*	- - - -
16.	GMR>Aβ42+*ptc*	+
17.	GMR>Aβ42+*Dl*	++
18.	GMR>Aβ42+*dTCF* ^*DN5*^	+++

A summary of the relative ability of these genetic modifiers to modify the Aβ42 phenotype. Number of (+)'s correlates to the strength of the rescue whereas (-) indicates the strength of the modifiers to enhance the neurodegenerative phenotype of Aβ42 phenotype.

We tested several candidates which included candidates of retinal determination (RD), negative regulators of eye development *homothorax* (*hth*), and members of highly conserved signaling pathways such as Wg, Dpp, Hh and N signaling pathways (Table 1). In comparison to the wild type compound eye (Figure 1A), the adult eye of GMR>Aβ42 is highly reduced in size with a slit like appearance and a glazed surface with black necrotic spots where extensive cell death has occurred (Figure 1B). Targeted misexpression of *tsh* with Aβ42 (GMR>Aβ42+*tsh*) resulted in significant rescue of this neurodegenerative phenotype (Figure 1C, Table 1). The frequency of the rescue phenotypes due to misexpression of the *tsh* was significantly higher. Nearly 75% of the GMR>Aβ42+*tsh* flies showed the strong rescue in the adult eye. The remaining 25% flies showed a weaker rescue phenotype. Members of the core retinal determination pathways, such as *ey* (GMR>Aβ42+*ey*; Figure 1D, Table 1), *eya* (GMR>Aβ42+*eya*; Figure 1E, Table 1), *so* (GMR>Aβ42+*so*; Figure 1F, Table 1), and *dac* (GMR>Aβ42+*dac*; Figure 1H, Table 1) did not show any significant rescue of the neurodegenerative phenotype of GMR>Aβ42. Furthermore, misexpression of *so* alone (GMR>*so*) results in reduced eye phenotype due to roughening of the anterior half of the eye and elimination of retinal tissue in the posterior half [71]. It has been shown that *G* *M* *R*>*e* *y* *a* had rough eyes [72]. Misexpression of *dac* in particular resulted in the most severe enhancement (Figure 1H) as compared to the other RD genes. The resulting GMR>Aβ42+*dac* phenotype was so strong that most of the progeny died as pharate and failed to hatch out of their pupal cases. Although GMR>Aβ42+*ey* and GMR>Aβ42+*eya* showed some overall increase in the size of the eye field, these eyes had a glazed surface morphology and lacked any ommatidia and pigment cells which allowed us to conclude that the neurodegenerative phenotype of GMR>Aβ42 was not rescued. It is known that Eya physically associates with So to perform a retinal differentiation function in the developing eye [51]. Targeted misexpression of both *eya* and *so* together (GMR>Aβ42+*e* *y* *a*+*s* *o*) did not result in rescue of the neurodegenerative phenotype (Figure 1G, Table 1). We also tested negative regulators of the eye such as *homothorax* (*hth*) (GMR>Aβ42+*hth*; Figure 1I, Table 1), which resulted in a subtle increase in the eye field but lacked any ommatidia. Misexpression of a dominant negative allele of *hth*, *hth* ^*ENR*^, (GMR>Aβ42+ *hth* ^*ENR*^), exhibited a significant increase in the eye field but these eyes had a neurogenic phenotype with increased numbers of bristles and lacked any pigment cells (Figure 1J, Table 1). Thus, there was only an increase in the number of cells with *no* ** *restoration* ** *of* ** *the* ** *eye* phenotype. Therefore, the members of the core retinal determination pathway and negative regulators of the eye did not have any major role in Aβ42 mediated neurodegeneration. We also tested the controls to verify our results (data not shown).

Furthermore, misexpression of *wg* (GMR>Aβ42+*wg*, Figure 1K, Table 1) resulted in a strong enhancement of the Aβ42 mediated neurodegenerative phenotype. All adult eyes were highly reduced in size with a glazed surface. The role of Wg signaling in Aβ42 mediated neurodegeneration was further validated using *shaggy* (*sgg*), an antagonist of Wg signaling [54]. Misexpression of *sgg* (GMR> Aβ42+*sgg*) resulted in a significant rescue, as evident from the increased size of the adult eye along with near wild-type looking ommatidia and bristles (Figure 1L; Table 1). Thus, modulating Wg signaling can modify Aβ42 mediated neurodegeneration. Interestingly, Dpp signaling is known to antagonize Wg signaling in the eye as well [37-39]. Misexpression of *dpp* (GMR>Aβ42+ *dpp*) resulted in a rescue (Figure 1M, Table 1), which is similar to what was observed upon blocking Wg signaling by misexpression of *sgg* (Figure 1L). The frequency of the rescue phenotypes in the adult eyes were siginificantly higher. Other candidates tested were optomotor blind (omb) (GMR> Aβ42+*omb*, Figure 1O; Table 1) and N (GMR> Aβ42+*N*, Figure 1N; Table 1), and they did not affect the neurodegenerative phenotype. Blocking Hh signaling pathway by using misexpression of *patched* (*ptc*) (GMR> Aβ42+*ptc*, Figure 1P, Table 1) resulted in a subtle increase in eye field with no rescue of neurodegeneration. Our screen resulted in identification of a homeotic gene, *tsh*, and members of Wg and Dpp signaling pathways as modifiers of Aβ42 mediated neurodegeneration. Surprisingly, *tsh* was able to provide one of the strongest rescues (Figure 1C; Table 1), and we therefore pursued to verify the neuroprotective role of *tsh* in Aβ42 mediated neurodegeneration. We also tested the controls to verify our results (data not shown).

## *tsh* Is a Genetic Modifier of Aβ42 Mediated Neurodegeneration

**Figure 2:**
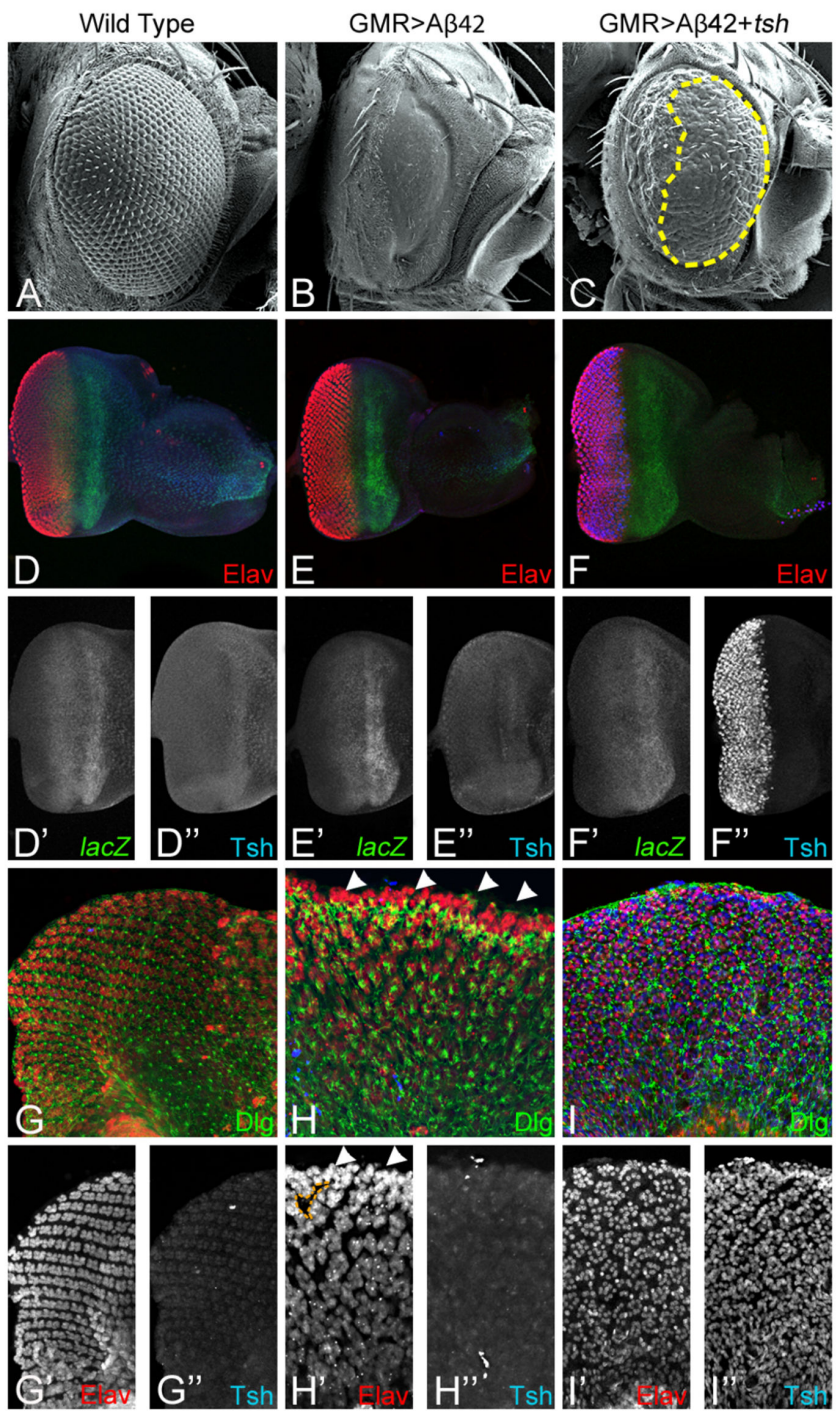
Ectopic induction of Tsh expression can rescue Aβ42 mediated neurodegeneration. (A-C) Scanning Electron Micrographs (SEM) of the adult *Drosophila* eye, (A) Wild type, (B) Misexpression of Aβ42 (GMR>Aβ42) in the differentiating photoreceptors of the developing eye results in a highly reduced eye due to lack of ommatidia and glazed surface due to extensive neurodegeneration. (C) Targeted misexpression of *tsh* and Aβ42 (GMR>Aβ42+*t* *s* *h*) in the differentiating photoreceptors of the eye leads to a significant rescue of the Aβ42 mediated neurodegenerative phenotype. (D-F) The *tsh* transcriptional status [marked by β-galactosidase reporter (*lacZ*; green channel)] and Tsh protein (Rabit Anti-Tsh antibody, blue channel) levels were tested in different genetic backgrounds in the developing eye-imaginal disc. Pan neural marker Elav (red channel) marks the neuronal fate. (D, D') *tsh* reporter is localized in bands both posterior and anterior to the morphogenic furrow (MF) in disc proper, (D") whereas Tsh protein is mainly restricted anterior to the MF in disc proper. (E, E') In the GMR>Aβ42 background similar compartmental patterns of *tsh* transcription (E, E") were seen. However, (E") Tsh expression is reduced as compared to (D") its expression in the wild type eye. (F- F") Targeted misexpression of *tsh* and Aβ42 (GMR>Aβ42+*t* *s* *h*) in the GMR domain of the eye-imaginal disc (F') show no deviation in *tsh* transcription from wild type, however (F") strong induction of Tsh expression is seen in the GMR domain. (G-I) In developing pupal retina Tsh protein and pan neural marker Elav were seen. The developing photoreceptors (marked by Elav in the red channel) in the pupal retina are arranged in a highly organized fashion (G, G') which is severely disrupted in the GMR>Aβ42 construct with fusion of ommatidia (marked by white arrow heads) and gaping holes (as marked by the yellow outline). (I, I') In the GMR>Aβ42+*t* *s* *h* pupal retina, ommatidial organization is restored as distinct ommatidial clusters are seen and no ommatidial fusion was seen (H') as compared to GMR>Aβ42. (G, G") Tsh is present in the developing ommatidia but did not show strong nuclear localization in the GMR>Aβ42 retina. (I, I") Strong induction of Tsh is present in both primary and secondary cells of the retina in the GMR>Aβ42+*t* *s* *h* construct. The magnification of (A-F) SEM micrographs of the adult eye is 180X, and confocal images of (D-F) the eye imaginal disc is 20X and (G-I) the pupal retina is 40X.

Targeted misexpression of full length *tsh* in the GMR>Aβ42 background (GMR> Aβ42+*tsh*; Figure 2C) significantly rescued the Aβ42 neurodegenerative phenotype of a highly reduced eye field with glazed appearance (Figure 2B). Even though a strong rescue was observed in the adult eyes of GMR>Aβ42+*tsh* flies (Figure 2C), the Aβ42 mediated neurodegenerative phenotype was not completely restored to the wild type adult eye phenotype (Figure 2A). Furthermore, we found that the rescue of neurodegeneration by *tsh* is spatial in nature as the *tsh* mediated rescue was restricted to the anterior half of the adult eye (Figure 2C; marked by yellow dotted line) whereas neurodegeneration still persists in the posterior half of the adult eye. In terms of the chronology of differentiation, the photoreceptors of the ommatidia close to the posterior margin are the oldest and the younger ommatidia are present in the anterior half of the adult eye. In the controls where GMR-GAL4 drive UAS-*tsh* transgene (GMR>*tsh*) resulted in a normal looking eye imaginal disc (Figure S1a), however, the adult eye was slightly reduced in size and lacking neurodegeneration on the posterior margin (Figure S1B). 

 We investigated the status of *tsh* transcription using a *lacZ* reporter [63] and Tsh protein levels in the GMR>Aβ42 background. During early eye development, both *tsh* ** *lacZ* and Tsh protein are expressed in the entire early eye primordium [46,73]. During retinal differentiation in the third instar eye imaginal disc, *tsh* ** *lacZ* (Figure 2D, D') and Tsh protein (Figure 2D, D") expression retracts anterior to the MF and Tsh is not localized in the differentiating photoreceptor neurons (Figure 2D-D"). However, the *tsh* ** *lacZ* domain exhibited less retraction as compared to the Tsh protein, probably due to perdurance of the lacZ protein, which serve as a reporter of *tsh* transcription. We found that *tsh* ** *lacZ* (Figure 2E, E') and Tsh protein (Figure 2E, E") levels were not affected in the GMR>Aβ42 background (Figure 2E, E"). Therefore, Tsh is absent in the developing neural retina when GMR>Aβ42 mediated neurodegeneration occurs. However, targeted misexpression of *tsh* with GMR>Aβ42 (GMR>Aβ42+ *tsh*) resulted in the rescue of Aβ42 mediated neurodegeneration and showed strong accumulation of Tsh protein in the differentiating photoreceptor neurons (Figure 2F, F"). Furthermore, we found that *tsh* ** *lacZ* expression was not induced in the differentiating neurons suggesting that Tsh protein, when misexpressed in a developing eye field, can provide neuroprotection. It also indicates that Tsh protein does not regulate its own transcription in the developing eye. 

Since the neurodegeneration phenotype of GMR>Aβ42 is progressive over the course of development, we analyzed Tsh levels in the pupal retina (Figure 2G-I). In the wild-type pupal retina, the nuclei of the differentiated neurons, marked by pan neural marker Elav (Figure 2G, G'), exhibited weak expression of Tsh (Figure 2G, G"). However, in the GMR>Aβ42 background, Tsh expression appears diffused probably due to the fact that photoreceptor nuclei are being disintegrated (Figure 2H, H' H"). Furthermore, there is clumping of the ommatidial nuclei in the pupal retina (Fig, 2H, H", marked by white arrow head), which results in holes in the pupal retina (Figure 2H', marked by yellow dotted line). However, in GMR>Aβ42+*tsh*, where robust expression of Tsh protein is observed, a significant rescue of the neurodegenerative phenotype is seen (Figure 2I, I', I"). The neuroprotective function of Tsh is evident from the regularly placed photoreceptor nuclei in the ommatidia and lack of holes in the retina of GMR>Aβ42+*tsh* (Figure 2I, I', I").

### Tsh can block induction of cell death

**Figure 3:**
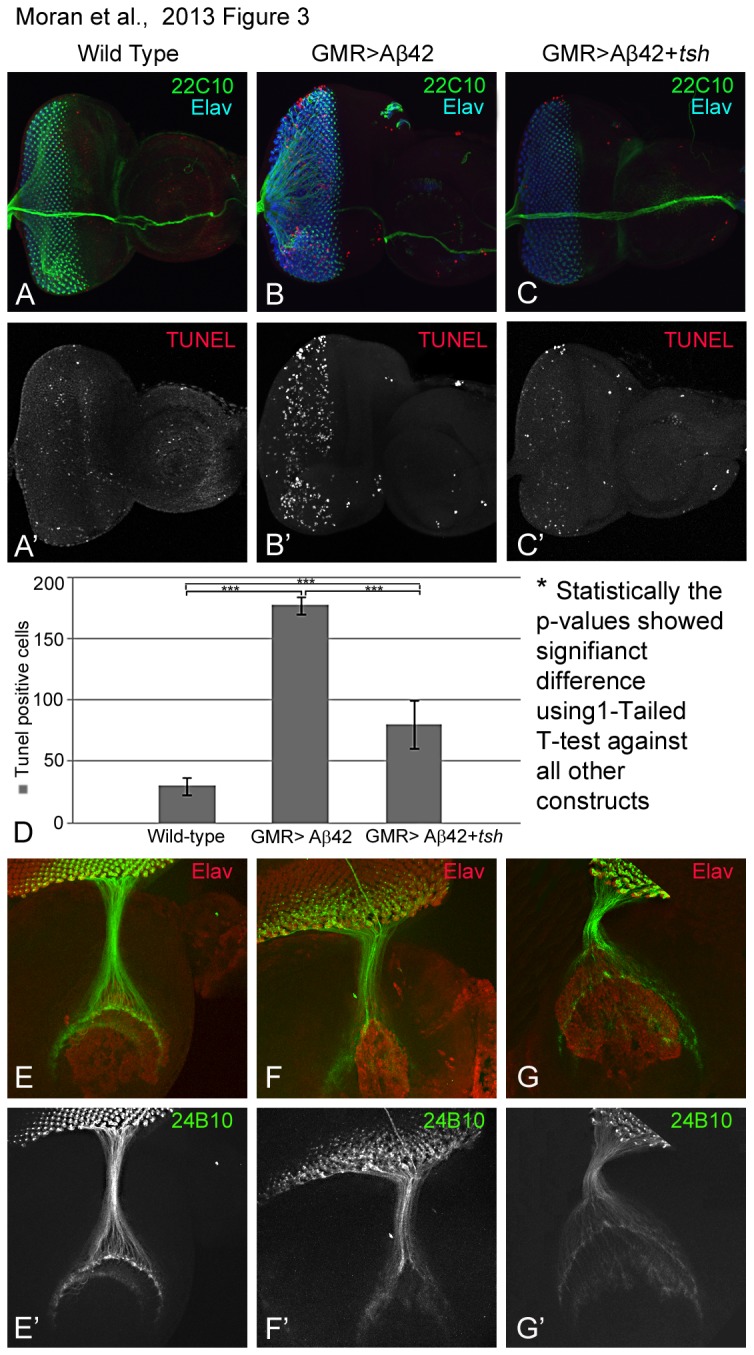
Ectopic induction of Tsh can rescue Aβ42 mediated neurodegeneration by blocking cell death. (A-C) Third instar eye-imaginal discs stained for 22C10 (marks the axonal sheath, in green channel), pan neural marker Elav (blue channel) and TUNEL that marks the nuclei of dying cells (red and split channels). (A, A') Wild-type eye imaginal disc showing random cell death in a few cells in the developing eye field, however, (B, B') the number of TUNEL positive dying cells nuclei increases dramatically in the GMR>Aβ42 background. (C, C') Targeted misexpression of *tsh* (GMR>Aβ42+*t* *s* *h*) significantly reduces the number of dying cell nuclei in the developing eye imaginal disc. (D) Quantitatively, the number of TUNEL cells have been counted and recorded with all five constructs shown. These phenotypes of enhancement of the neurodegenerative phenotype and rescue, based on the number of TUNEL positive cells, are significant as seen by the calculation of P-values based on the one-tailed t-test using Microsoft Excel 2010. Note that the number of dying cells increased more than three folds in the GMR>Aβ42 background as compared to the wild-type eye imaginal disc. The number of dying cells in GMR>Aβ42+*t* *s* *h* background is reduced to half as compared to GMR>Aβ42. Although the number of dying cells in GMR>Aβ42+*t* *s* *h* background it is still more than the wild-type eye disc. (E-G) Photoreceptor cells projections in third instar larva visualized using Chaoptin (MAb24B10, green channel) [65] staining. MAb24B10 marks the retinal axons from the neural retina to the brain, and proneural marker Elav (red channel). (E') In the wild type larva, retinal axons projection pattern from the photoreceptors in the retina to the optic lobes in the brain. Note that ommatidial axonal bundle from eye field contacts the brain at two locations in a highly organized fashion in the wild type. (E', F') Misexpression of (F') Aβ42 (GMR>Aβ42) results in aberrant retinal axon targeting from the neural retina to the brain (E") as compared to the wild type. (G') Targeted misexpression of *tsh* in the Aβ42 background (GMR>Aβ42+ *tsh*) results in a strong restoration of retinal axonal targeting. The magnification of confocal images of (A-C) the Eye-antennal imaginal disc is 20X, and (E-G) the retinal axon is 60X.

To further validate our hypothesis of a neuroprotective role of *tsh* in Aβ42 mediated neurodegeneration, we investigated the rate of cell death using a TUNEL staining approach. TUNEL staining marks the fragmented ends of DNA of the dying cells nuclei [67,68]. In comparison to the wild-type eye imaginal disc, which exhibits a few cells undergoing cell death based on few TUNEL positive cells (Figure 3A, A'), the GMR>Aβ42 eye imaginal disc exhibits a significantly higher number of TUNEL positive cells (Figure 3B, B', D). A higher number of TUNEL positive cells can explain the highly reduced eye size of the GMR>Aβ42 adult eye [26]. We counted the number of dying cells in the wild type as well as GMR>Aβ42 eye disc and found that the number of dying cells increases by more than three fold in the GMR>Aβ42 disc as compared to the wild-type eye (Figure 3D). Furthermore, targeted misexpression of *tsh* with GMR>Aβ42 (GMR>Aβ42+*tsh*) results in a significant rescue of the neurodegenerative phenotype where the number of TUNEL positive dying cells was reduced to half the number of cells undergoing cell death in the GMR>Aβ42 eye disc (Figure 3C, C', D). These results further validate our hypothesis that *tsh* can provide neuroprotection against GMR>Aβ42 mediated neurodegeneration. 

### Tsh can rescue axonal targeting defects

During development of the *Drosophila* visual system connections are generated from the neural retina to the brain by means of axonal targeting [74,75]. The differentiating neurons send out axons that lead by their growth cones and precisely trace their appropriate synaptic targets. In the developing eye imaginal disc, each differentiated photoreceptor neuron send axons[74]. The axons from the photoreceptors of ommatidia fasciculate together to form an ommatidial bundle. The ommatidial bundle pierces through the basement membrane of the eye disc and then extends to the posterior edge of the eye disc, and through the optic stalk innervates the different layers of the brain [76,77]. The axons from photoreceptors (PRs) 1-6 terminate in the lamina whereas PR7-PR8 terminates in separate layer of medulla after passing through lamina. The retinal axons can be marked by Chaoptin (MAb24B10) [65]. In the wild-type eye imaginal disc MAb24B10 marks the axons which innervate the different layers of the optic lobe of the brain (Figure 3E, E'). However, in the GMR>Aβ42 eye imaginal disc, the retinal axon targeting becomes impaired as we can no longer observe axons innervate properly in the optic lobes of the brain (Figure 3F, F'). Misexpression of *tsh* in the GMR>Aβ42 background (GMR>Aβ42+*tsh*) can not only restore the size of the eye field but can also significantly restore the retinal axon targeting phenotype (Figure 3 G, G'). 

### PLDLS domain is a negative regulator of the neuroprotective function of Tsh

**Figure 4:**
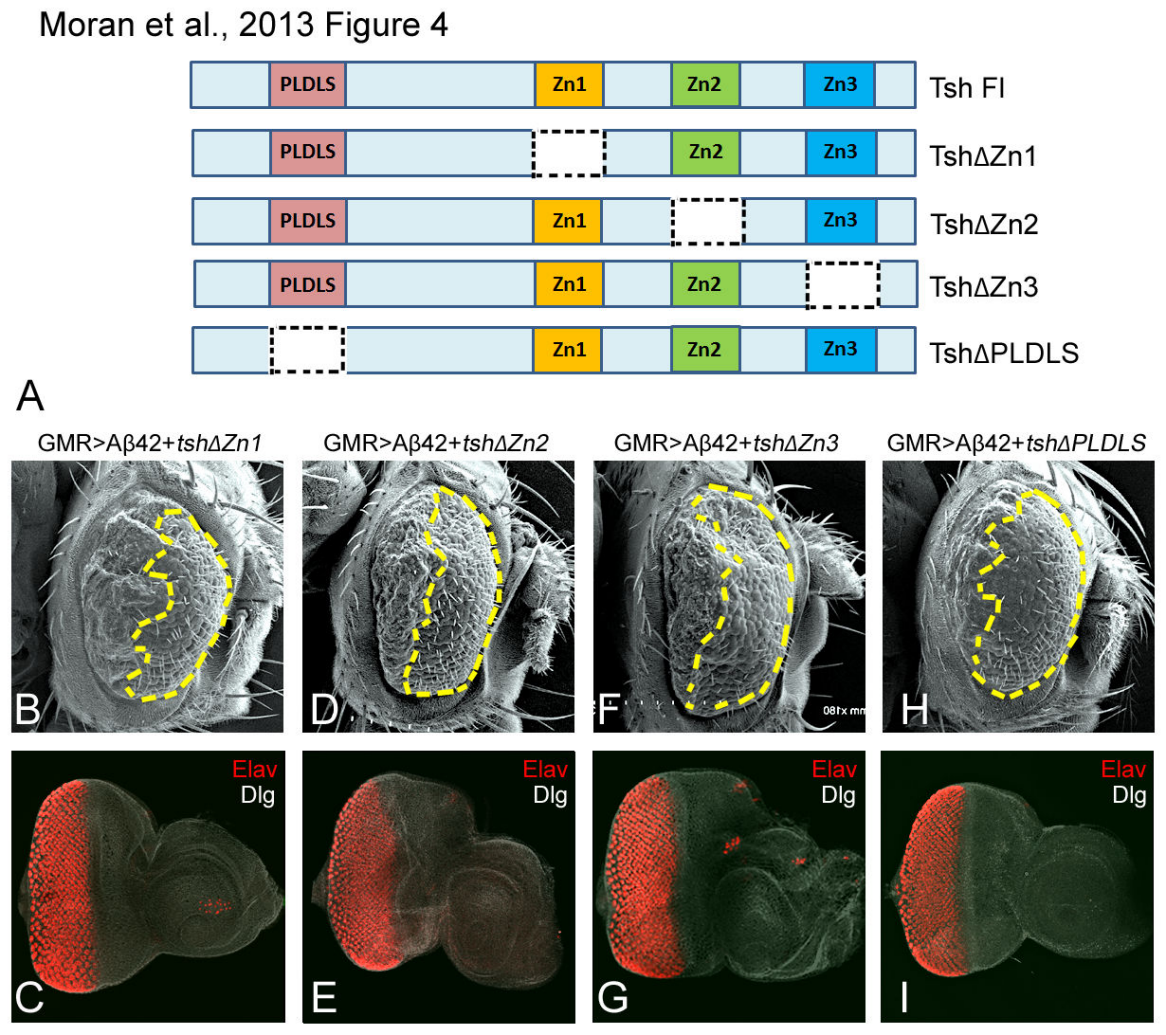
PLDLS domain of Tsh acts as a suppressor of its neuroprotective function. (A) A cartoon depicting full length Tsh protein and various truncated constructs [49] used in this study. The full length Tsh protein consists of a PLDLS domain and three DNA binding Zinc finger domains. (B-E) Scanning electron micrographs (SEM) of the adult eyes generated by targeted misexpression of various deletion constructs of *tsh* in the GMR>Aβ42 background to test their requirement in its neuroprotective function. A membrane specific marker Disc large (Dlg, white channel), was used to mark the outline of cells and pan neural marker Elav (red channel) marks nuclei of the photoreceptor neurons. Targeted misexpression of Aβ42 and deletion constructs of *tsh* lacking the (B, C) first zinc finger domain (GMR>Aβ42+*t* *s* *h*∆Zn1), (D, E) second zinc finger domain (GMR>Aβ42+*t* *s* *h*∆Zn2), and (F, G) third zinc finger domain (GMR>Aβ42+*t* *s* *h*∆Zn3) resulted in a significant rescue of the Aβ42 mediated neurodegeneration in the imaginal disc and the adult eye, respectively. Note that the extent of rescue by deletion constructs lacking various Zn finger domains is comparable to the full length Tsh protein. (H, I) Targeted misexpression of Aβ42 and deletion construct of the PLDLS domain (GMR>Aβ42+*t* *s* *h*∆PLDLS) results in a significantly stronger rescue both in the (H) eye imaginal disc and the (H) adult eye. Note that this rescue is stronger than the one seen with (Figure 2C) full length Tsh protein. The magnification of (B, D, F, H) the adult eye SEM image is 180X, and (C, E, G, I) the eye-antennal imaginal disc is 20X.

*tsh* encodes a C_2_H_2_ zinc finger transcription factor protein which consists of a PLDLS domain and three zinc finger domains [19,44-49,73,78]. We performed a structure function analysis to determine the role of various domains of the Tsh protein in its neuroprotective function by utilizing various deletion constructs of *tsh*. These constructs were generated by individually removing each domain from the full length Tsh protein [49]. We used these constructs to misexpress truncated Tsh protein with Aβ42 and then screen for the domain required for its neuroprotective function. We found that misexpression of truncated Tsh lacking the Zn1 domain (GMR>Aβ42+*t* *s* *h*∆Zn1) (Figure 4A, B, C), Zn2 domain (GMR>Aβ42+*t* *s* *h*∆Zn2) (Figure 4A, D, E) or Zn3 domains (GMR>Aβ42+*t* *s* *h*∆Zn3) (Figure 4A, F, G) was able to rescue the GMR>Aβ42 phenotype as seen in the eye antennal imaginal disc (Figure 4C, E, G) as well as the adult eye (Figure 4 B, D, F), respectively. Interestingly, the rescue by truncated *tsh* lacking these three zinc finger domains were comparable to the full length *tsh* as seen in (Figure 1C, 2C, F). Therefore, removing zinc finger domains does not affect the neuroprotective function of *tsh*. Misexpression of truncated Tsh lacking the PLDLS domain (GMR>Aβ42+*t* *s* *h*∆PLDLS) showed a stronger rescue of Aβ42 mediated neurodegeneration in the eye imaginal disc and the adult eye (Figure 4A, H, I). Interestingly, the rescue by truncated Tsh lacking the PLDLS domain was much stronger than the rescue by the full length Tsh, suggesting that the PLDLS domain acts as a suppressor of the neuroprotective function of *tsh*. Our results strongly suggested that DNA binding domains of *tsh* such as Zn1, Zn2 and Zn3 are either functionally redundant or play no significant role in the neuroprotective function of *t* *s* *h*.

### *tio* can rescue Aβ42 mediated neurodegeneration

**Figure 5:**
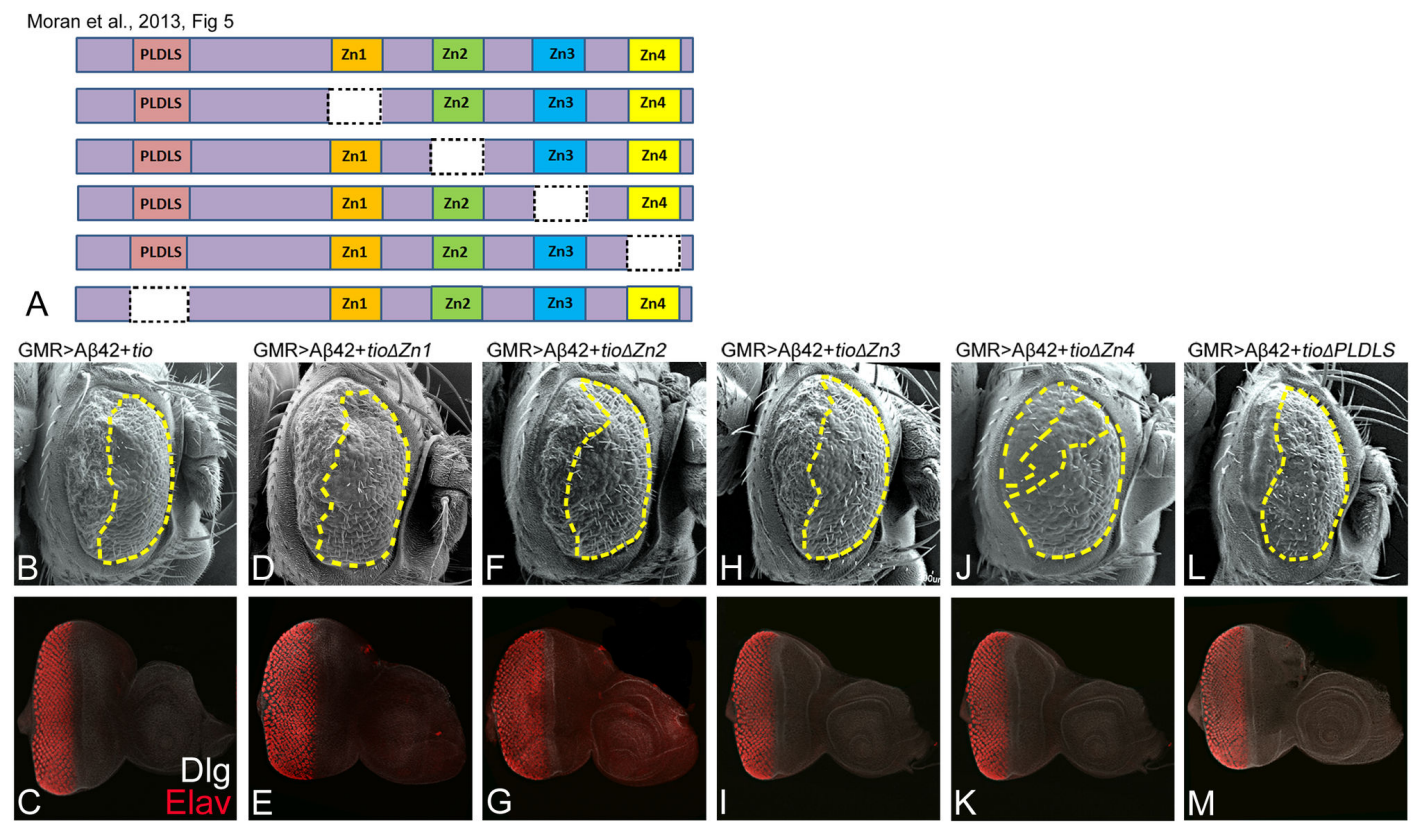
A paralog of *tsh*, *tio*, also exhibits a neuroprotective function. (A) A cartoon depicting full length type Tio protein and various truncated constructs used in this study [49]. The full length Tio protein consists of a PLDLS domain and four DNA binding Zinc finger domains [49]. A membrane specific marker Disc large (Dlg, white channel) was used to mark the outline of cells and pan neural marker Elav (red channel) marks nuclei of the photoreceptor neurons. Targeted misexpression of Aβ42 and deletion constructs of the (B, C) first zinc finger domain (GMR>Aβ42+*t* *i* *o*∆Zn1), (D, E) second zinc finger domain (GMR>Aβ42+*t* *i* *o*∆Zn2), (F, G) third zinc finger domain (GMR>Aβ42+*t* *i* *o*∆Zn3) resulted in the significant rescue of Aβ42 mediated neurodegeneration in the adult eye, which is comparable to the full length Tio protein. However, targeted misexpression of (J, K) the fourth zinc finger domain (GMR>Aβ42+*t* *i* *o*∆Zn4), and (L, M) deletion constructs of the PLDLS domain (GMR>Aβ42+*t* *i* *o*∆PLDLS) resulted in a significantly stronger rescue. Note that this rescue is stronger than the one seen with (Figure 2C) full length Tio protein. The magnification of (B, D, F, H, J, L) the adult eye SEM micrographs is 180X and (C, E, G, I, K, M) the eye-antennal imaginal disc confocal images is 20X.

*tsh* shares a regulatory relationship with its paralog - *tio* [44,47,49], which is expressed in a similar pattern and encodes a protein with four zinc finger domains and a N-terminal CtBP domain (Figure 5A). Targeted misexpression of full length *tio* in the GMR>Aβ42 background (GMR>Aβ42+*tio*) showed a significant rescue of the Aβ42 mediated neurodegeneration phenotype as seen in the (Figure 5B) adult and (Figure 5C) the imaginal disc. We observed a significantly stronger rescue in terms of increased eye size with normal looking, regularly arranged, ommatidia and little or no patches of necrosis in the anterior region (Figure 5B). Furthermore, the rescue of Aβ42 mediated neurodegeneration by *tio* is similar to *tsh* (Figure 2C). 

To determine the role each domain of *tio* plays in its neuroprotective function, we carried out a structure function analysis using deletion constructs of *tio* generated by individually removing each domain from the full length protein [49]. We used these constructs to misexpress truncated Tio protein with Aβ42 and then screened for the domain required for its neuroprotective function. We found that misexpression of truncated Tio lacking the Zn1 domain (GMR>Aβ42+*t* *i* *o*∆Zn1) (Figure 5A, D, E), Zn2 domain (GMR>Aβ42+*t* *i* *o*∆Zn2) (Figure 5A, F, G) or Zn3 domain (GMR>Aβ42+*t* *i* *o*∆Zn3) (Figure 5A, H, I) was able to rescue the GMR>Aβ42 phenotype as seen in the eye antennal imaginal disc (Figure 5 E, G, I) as well as the adult eye (Figure 5 D, F, H), respectively. Interestingly, the rescues by truncated *tio* lacking Zn1 or Zn2 or Zn3 domains were comparable to the full length *tio* as seen in (Figure 5A,B, C), suggesting that these domains are not required for the neuroprotective function of *tio*. We found that misexpression of truncated Tio lacking the Zn4 domain (GMR>Aβ42+*t* *s* *h*∆Zn4; Figure 5A, J, K) or PLDLS domain (GMR>Aβ42+*t* *s* *h*∆PLDLS; Figure 5 A, L, M) domain showed a significant rescue of Aβ42 mediated neurodegeneration as seen in the adult eye (Figure 5A, J, L) and the eye imaginal disc (Figure 5A, K, M), respectively. Therefore, removal of the Zn4 (Figure 5A, J, K) or the PLDLS (Figure 5A, L, M) domain from the full length Tio increased the intensity of rescue as compared to the full length *tio* (Figure 5A, B, C), suggesting that the Zn4 and the PLDLS domain act as suppressor of the neuroprotective function of *tio*. 

## Discussion

 Several signaling pathways may play a role in GMR>Aβ42 mediated neurodegeneration. Generally, accumulation of GMR>Aβ42 plaques triggers some aberrant signaling response which finally triggers abnormal signaling leading to generation of stress in the neurons and finally culminating in the death of the neurons [2-7,26,79]. One of the most important facets of this process is to understand the downstream targets of amyloid beta mediated neurodegenerative response. A genome wide forward genetic screen can be labor intensive and therefore, we employed a candidate gene approach where we picked up the candidates of the various signaling pathways and tested them individually. Our candidate genes approach for the forward genetic screen resulted in identification of homeotic gene *tsh* as a neuroprotective agent. Interestingly, *tsh* has been shown to be involved in patterning, growth and retinal development [31,45,46,73,78]. However, its role as a neuroprotective agent has not been fully understood.

### Tsh has a neuroprotective role in Aβ42 mediated neurodegeneration

Our data suggests that misexpression of Tsh in the differentiating photoreceptor neurons of the fly retina can rescue the neurodegenerative phenotype of GMR>Aβ42 mediated neurodegeneration. We found that the neuroprotective function of *tsh* is mediated through prevention of induction of cell death (Figure 3). Earlier, we showed that GMR>Aβ42 mediated cell death is both caspase dependent and caspase independent [26]. Since *tsh* can significantly restore the neurodegenerative phenotype (Figure 2C), it is expected that *tsh* mediated rescue might affect either one or both of them significantly. It will be interesting to look for the mechanism by which *tsh* can prevent induction of neurodegeneration in the GMR>Aβ42 background. Tsh is a homeotic gene and is involved in several signaling pathways to regulate patterning and growth [19,31,45,46,73,78]. Tsh is known to be involved in regulating retinal development in *Drosophila* and has the capability to induce ectopic eyes [31]. Since our disease model is restricted to the retina of the fly, we wanted to test if the neuroprotective function of Tsh is mediated through its role in the retinal differentiation pathway [19,31,46,48,78].

### Neuroprotective function of Tsh is independent of RD gene function

**Figure 6:**
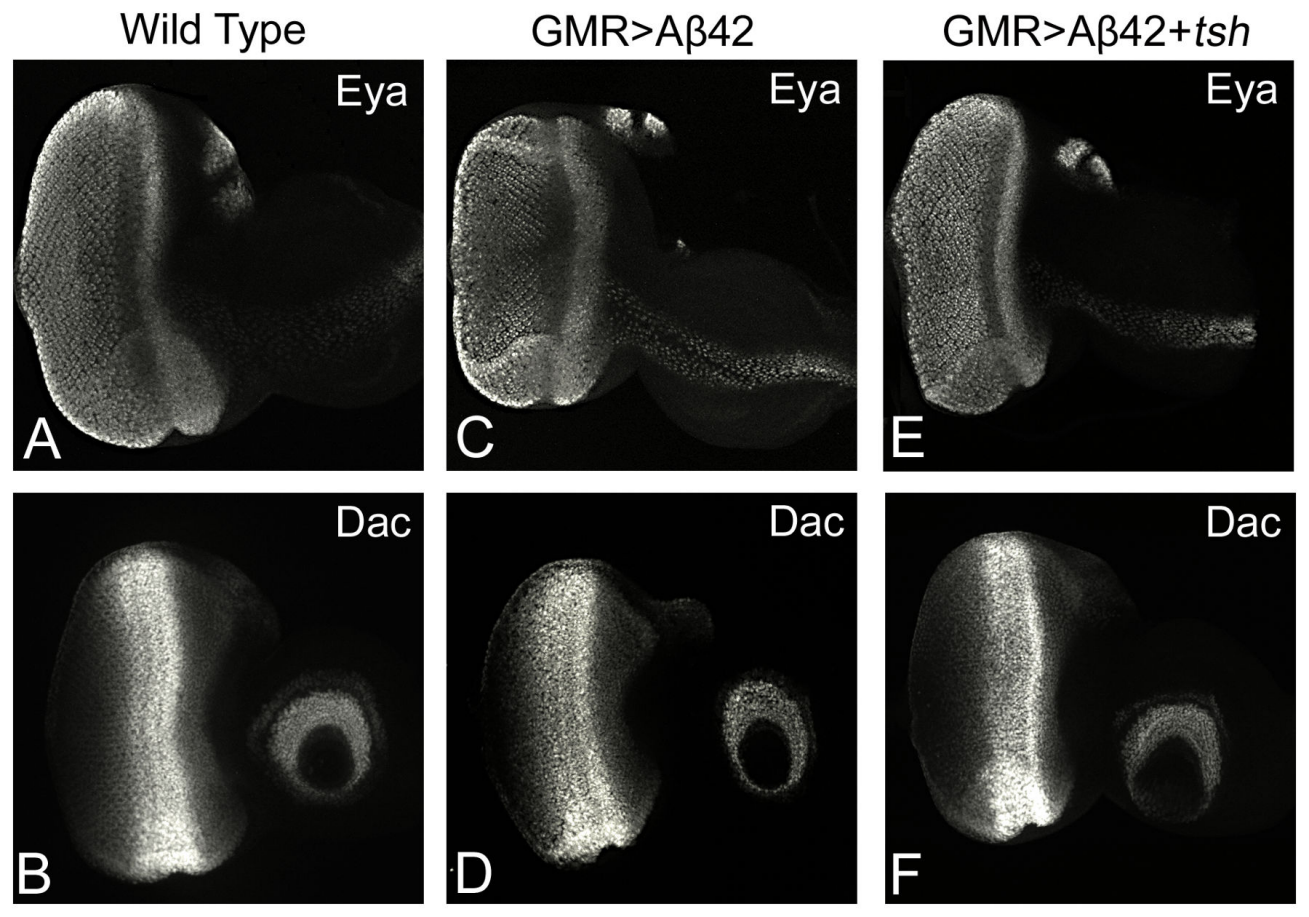
Tsh neuroprotective function in Aβ42 mediated neurodegeneration is independent of retinal determination gene function. (A, C, E) Expressison of Eyes Absent (Eya), a member of the RD gene machinery, in third instar eye imaginal discs. (A) Eya is expressed in the differentiating photoreceptors and anterior to the MF. (C, E) Eya expression in the (C) GMR>Aβ42 and (E) targeted misexpression of Aβ42 and *tsh* (GMR>Aβ42+ *tsh*) backgrounds does not deviate from (B) wild type Eya expression. (B, D, F) Expressison of Dachshund (Dac), a member of the RD gene machinery, in third instar eye imaginal discs (B) Wild type Dac expression is induced prominently along the MF as well as in the antennal region. (D, F) Dac expression in the (C) GMR>Aβ42 and (E) targeted misexpression of Aβ42 and *tsh* (GMR>Aβ42+ *tsh*) background does not deviate from (B) wild type Dac expression. Note that RD gene expression is not affected in the *tsh* mediated rescue of GMR>Aβ42 neurodegenerative phenotype. The magnification of all the confocal images of eye-antennal imaginal disc is 20X.

It has been shown that *tsh* can induce *eya* and *so* to form an ectopic eye in the antenna [31]. Based on these results, *tsh* was assigned to the category of genes which are involved in eye development even though they do not belong to the core retinal determination pathway[19]. We investigated the role of RD genes in the neuroprotective function of *tsh* by testing levels of the RD genes *eya* and *dac* in the third instar eye imaginal disc. Eya, a tyrosine phosphatase, is involved in the retinal differentiation process and is expressed both in the differentiating photoreceptor neurons of the neural retina as well as the retinal precursor cells anterior to the MF (Figure 6A, A') [17,18,30]. In the GMR>Aβ42 background third instar eye imaginal disc we found that Eya expression is not affected (Figure 6B) and in the GMR>Aβ42+*tsh* background, we did not see any ectopic induction or increased levels of Eya in the differentiating neurons (Figure 6C). We also investigated levels of *dac*, another RD gene[52], which is expressed in two different domains one anterior to the MF and another posterior to the MF (Figure 6D). In the GMR>Aβ42 (Figure 6E) as well as the GMR>Aβ42+*tsh* background (Figure 6F), we did not find any change in the levels of Dac expression. Our data strongly suggests that the neuroprotective role of Tsh is independent of its role in RD gene function. This data is consistent with the result from the genetic screen where we found that increasing levels of the RD gene members did not affect the GMR>Aβ42 mediated neurodegenerative phenotype (Figure 1, Table 1). Therefore, the neuroprotective role of *tsh* is a novel function of this genetic locus. 

### Tsh neuroprotective role is downstream of FE65 mediated regulation of APP cleavage

Aβ42, the major component of amyloid plaques, is a by-product of the improper cleavage of Amyloid Precursor Protein (APP) protein [80]. Tsh has been shown to be involved in processing of Amyloid Precursor Protein (APP) cleavage. It has been shown that APP can bind to FE65 protein and this complex can regulate gene expression. FE65 is an adaptor protein with two phosphotyrosine binding (PTB) domains and a single WW domain [81,82]. In a yeast two hybrid screen using the PTB domain of FE65 protein as a bait, Tsh was identified as an FE65 interacting protein in the neurons [82]. The co-immunprecipitation studies showed a direct interaction of FE65 and Tsh 3, one of the vertebrate homologs of Tsh, with the promoter region of Caspase 4 [82]. It was demonstrated that Tsh can work in a protein complex to trigger cell death in neuritic plaques of AD. Interestingly, in *Drosophila* there is a no homolog of FE65 present. Therefore, Tsh cannot physically associate with FE65. Therefore, in our studies the neuroprotective function of *tsh* is due to prevention of neurodegeneration caused by accumulation of human Aβ42 in the system. The mechanism by which Tsh can prevent neurodegeneration is yet to be fully understood.

### Whether Tsh blocks or delays the onset of neurodegeneration

Our studies also raised an interesting question of whether Tsh mediated neuroprotective phenotype which has spatial component as the rescues were restricted to the anterior half of the adult eye (Figure 2C), has some temporal component. There were no examples of rescues in the posterior part of the adult eye. In the *Drosophila* eye, differentiation initiates from the posterior margin of the developing eye imaginal disc and moves in a synchronous fashion towards the anterior margin of the eye field. Therefore, in terms of chronology, the ommatidia on the posterior margin are older in comparison to the ones towards the anterior margin of the eye imaginal disc [21,22]. Interestingly, targeted coexpression of *tsh* with Aβ42 (GMR> Aβ42+*tsh*) results in significant rescue of the eye but the rescue is restricted to the anterior half of the adult eye (Figure 2C). It raises an interesting possibility that either it takes time for the *tsh* levels to build up and as a result it cannot rescue neurodegeneration in the older retinal neurons of the posterior half of the adult eye, or *tsh* alone can only delay the onset of neurodegeneration in the neural retina and as a result the adult eye exhibits rescue in the anterior half. We therefore analyzed the adult eye phenotype of GMR>Aβ42+*tsh* adults of different ages. We found that the size of the eye field reduces with age. In comparison to the freshly eclosed one day old fly, the thirty day old fly eye is reduced along with increased loss of pigment cells and more necrotic spots (Figure S2). 

We also investigated whether a paralog of *tsh*, *tio*, can rescue the neurodegenerative phenotype and we found functional redundancy between *tsh* and *tio*. The different functional domains of Tsh and Tio tested for the neuroprotective function showed that PLDLS domain act as suppressor in both Tio and Tsh (Figure 4, 5). These results further validate that the functional redundancy observed between Tsh and Tio during retinal development [44,49] may also hold true in terms of their neuroprotective functions. Our studies also suggest that Tsh and Tio might play a role in vertebrates in protecting the neural retina from Aβ42 mediated neurodegeneration as well. Further, the neuroprotective function of Tsh and Tio can be extrapolated in other neuronal population too as well as in other organisms.

## Supporting Information

Figure S1 **Misexpression of *tsh* (GMR>*tsh*) in the differentiating photoreceptor neurons of the developing retina.** (A) Eye antennal disc, and (B) Adult eye. Note that adult eye exhibits slight reduction on the posterior margin. The magnification of (A) confocal image of the eye -antennal imaginal disc is 20X and (B) the SEM micrograph of the adult eye is 180X.
(TIF)

Figure S2 **Targeted misexpression of *tsh* can delay the onset of neurodegeneration.** Adult flies of genotype GMR>Aβ42+*t* *s* *h* were collected and staged. The adult eye phenotypes of GMR>Aβ42+*t* *s* *h* on days (A) one, (B) ten, (C) twenty, and (D) thirty shows progressive reduction in the eye size. The magnification of brightfield images of the adult eyes is 10X.
(TIF)
