# Supplementary figures and images for: Homeotic Gene teashirt (tsh) Has a Neuroprotective Function in Amyloid-Beta 42 Mediated Neurodegeneration
Source: PLoS One. 2013 Nov 25;8(11):e80829. doi: 10.1371/journal.pone.0080829 (PMC3840013; doi:10.1371/journal.pone.0080829)

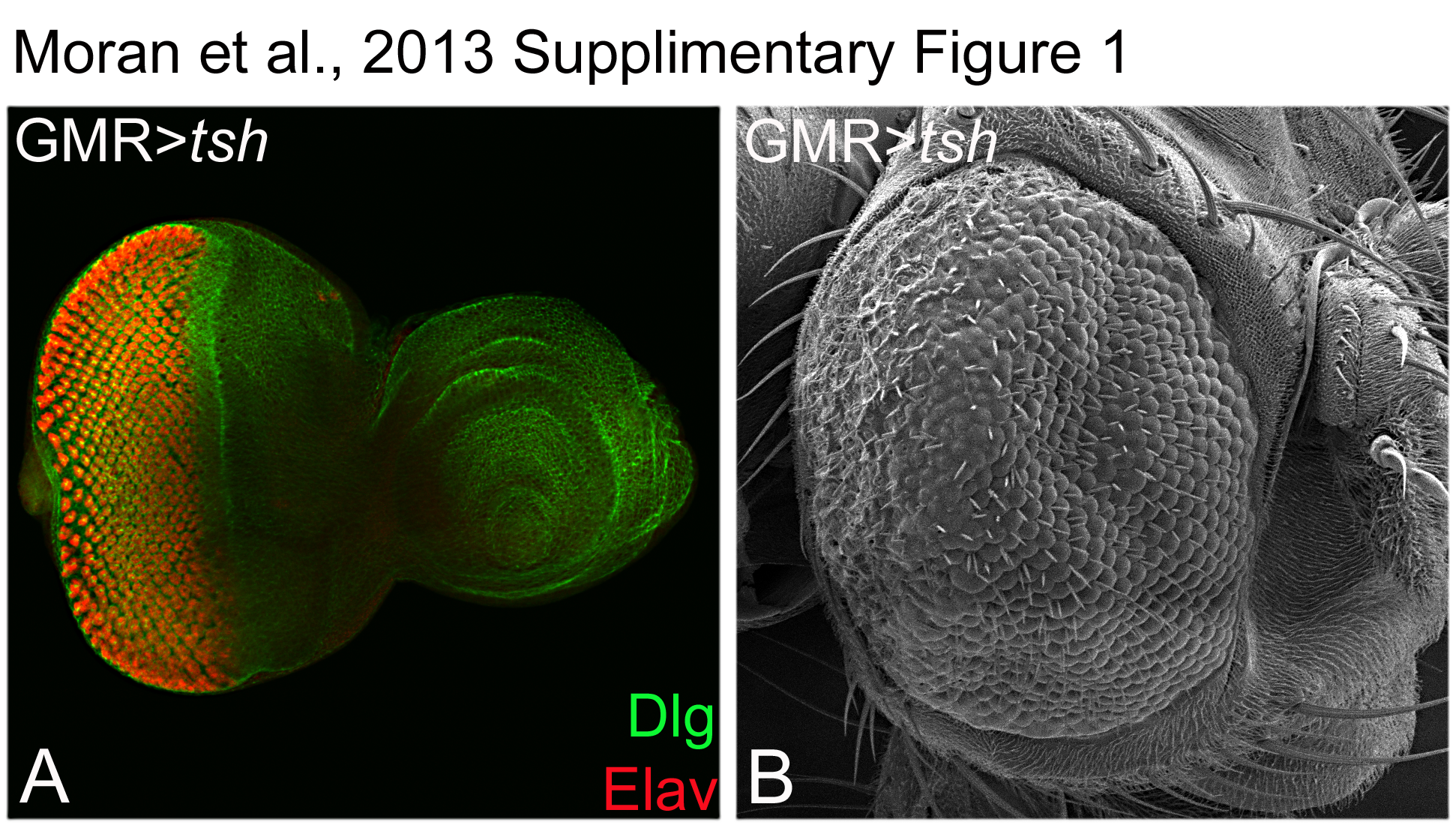

Supplement: Figure S1 — Misexpression of tsh (GMR>tsh) in the differentiating photoreceptor neurons of the developing retina. (A) Eye antennal disc, and (B) Adult eye. Note that adult eye exhibits slight reduction on the posterior margin. The magnification of (A) confocal image of the eye -antennal imaginal disc is 20X and (B) the SEM micrograph of the adult eye is 180X. (TIF) [file pone.0080829.s001.tif]

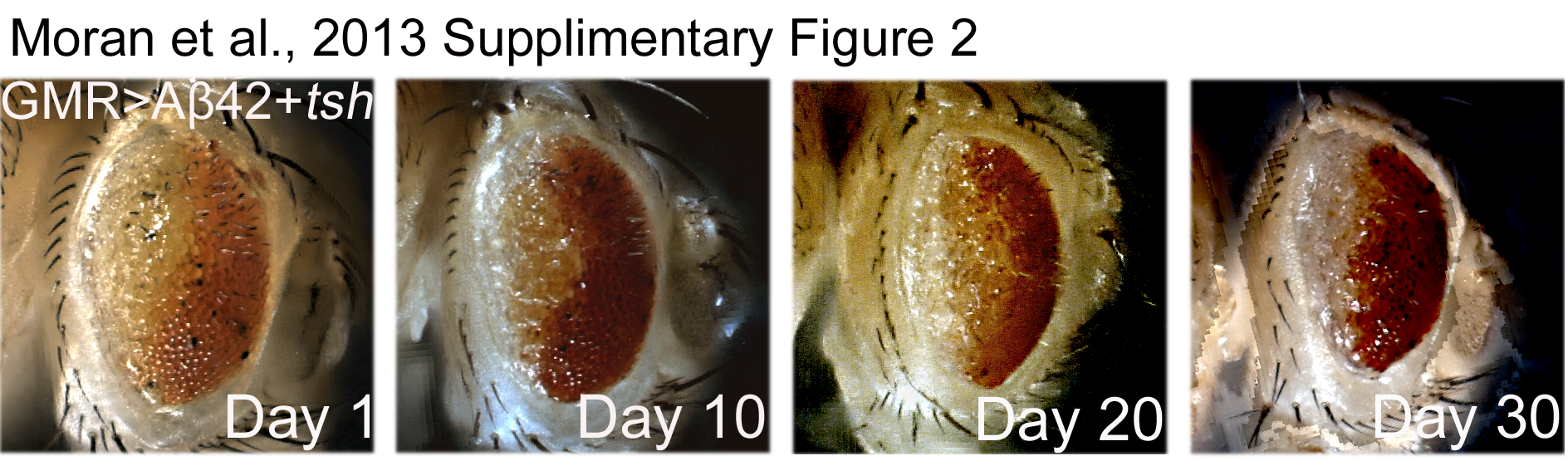

Supplement: Figure S2 — Targeted misexpression of tsh can delay the onset of neurodegeneration. Adult flies of genotype GMR>Aβ42+t s h were collected and staged. The adult eye phenotypes of GMR>Aβ42+t s h on days (A) one, (B) ten, (C) twenty, and (D) thirty shows progressive reduction in the eye size. The magnification of brightfield images of the adult eyes is 10X. (TIF) [file pone.0080829.s002.tif]
